# Adverse childhood experiences and cognitive function in adulthood: examining the roles of depressive symptoms and inflammation in a prospective cohort study

**DOI:** 10.1007/s00127-022-02315-w

**Published:** 2022-06-26

**Authors:** Elaine Lowry, Amy McInerney, Norbert Schmitz, Sonya S. Deschênes

**Affiliations:** 1 UCD School of Psychology, University College Dublin, Dublin, Ireland; 2 Department of Psychiatry, McGill University, Montreal, QC Canada; 3 Department of Population-Based Medicine, University of Tübingen, Tübingen, Germany

**Keywords:** Adverse childhood experiences, Cognitive function, Depression, Inflammation

## Abstract

**Purpose:**

Adverse childhood experiences (ACEs) have been associated with cognitive decline in adulthood. However, the underlying mechanisms implicated remain unclear. This study investigated depressive symptoms and systemic inflammation as potential mediators of the association between ACEs and later cognitive function.

**Methods:**

Participants were adults aged 50 + from the English Longitudinal Study of Ageing (*N* = 3029; 54.8% female). Measures included self-reported ACEs at wave 3 (2006–2007), C-reactive protein (CRP) and depressive symptoms at wave 4 (2008–2009), and cognitive function at waves 3 and 7 (2014–2015). Mediation analyses examined the direct associations between ACEs and cognitive function at wave 7 and the indirect associations via depressive symptoms and CRP at wave 4. In a first set of analyses, models were adjusted for sociodemographic factors and baseline cognitive function. In a second set of analyses, models were additionally adjusted for BMI and health behaviours (*n* = 1915).

**Results:**

Cumulative ACEs exposure positively predicted depressive symptoms (*b* = 0.184, s.e*.* = 0.034, *p* < .001), which in turn predicted poorer cognitive function at wave 7 (*b* =  − 0.035, s.e*.* = 0.008, *p* < .001). ACEs also positively predicted systemic inflammation as measured by CRP (*b* = 0.031, s.e*.* = 0.01, *p* = 0.0016). However, CRP did not mediate the association between ACEs and later cognitive function (*b* =  − 0.0002, 95% CI: − 0.002, 0.002).

**Conclusion:**

These findings suggest that ACEs may be related to cognitive decline partly via depressive symptoms and corroborate prior research linking ACEs with systemic inflammation in adulthood.

**Supplementary Information:**

The online version contains supplementary material available at 10.1007/s00127-022-02315-w.

## Introduction

Cognitive decline is a major public health issue. By 2050, the global population aged 65 years and above is projected to double, with those aged over 80 expected to triple [1]. Cognitive decline is associated with a reduced capacity for activities of daily living [2] and increased risk of hospitalisation, nursing home admission, and mortality [3]. The aetiology of accelerated cognitive decline may begin in early life. Adverse childhood experiences (ACEs) have been associated with adult cognitive deficits, particularly in memory, processing speed, and executive function [4, 5] including during later life [6–8]. ACEs include child maltreatment (e.g. abuse and neglect) and household dysfunction (e.g. violence in the home, parental addiction, mental illness, or institutionalisation) [9]. A recent study, using data from the English Longitudinal Study of Ageing (ELSA), demonstrated a mild association between ACEs and memory decline over ten years from middle to older age [10]. Memory, processing speed, and executive function deficits have also been associated with ACE exposure among middle-aged adults with depression [11, 12]. Child maltreatment has been identified as a risk factor for more severe and long-lasting cognitive impairment in adults with major depressive disorder [13]. Although ACEs are associated with accelerated cognitive decline, the underlying mechanisms implicated remain unclear. A greater understanding of these mechanisms is needed to better target preventative and mitigation efforts.

A growing body of evidence suggests that depression may play an indirect role in the mechanism underlying the association between ACEs and cognitive decline. Depression is associated with deficits in executive function, processing speed, memory, and attention [14, 15]. Late-life depression has also been linked to an increased risk of dementia [16, 17]. Kuring et al. [18] identified a strong association between depression and later all-cause dementia. However, the authors note that while depression may be a prodrome of dementia, additional longitudinal research is needed to investigate a potential causal link.

ACE exposure is also linked to adult depression. In a seminal study, Felitti et al. [11] found that reporting four or more ACEs increased the risk of adult depression by 4.6-fold compared to reporting no ACEs. A recent meta-analysis found that adults reporting any form of child maltreatment were 2.5 times more likely to develop depression than those reporting none [19]. Though limited, the available evidence among older adults suggests that ACEs, particularly abuse, are strongly associated with an elevated risk of later-life depressive symptoms [20–22]. A study from 2020, using ELSA data, linked cumulative ACE exposure to higher depressive symptoms in older adults [23]. Depressive symptoms were previously shown to mediate the association between childhood abuse and poor cognitive functioning in a middle-aged sample [24]. However, temporal precedence may not have been sufficiently established given the short interval between assessment of depression and cognitive performance in this study (*M* = 20 months). Therefore, further long-term longitudinal evidence is required to better investigate the role of depression in the ACEs-cognitive decline association.

Systemic inflammation presents another potential pathway in the ACEs-cognitive decline association. Cross-sectional studies of older adults have observed an association between higher C-reactive protein (CRP) levels, an acute-phase protein used to assess systemic inflammation [25], and poorer performance on tests of memory [26, 27] and executive function [28]. In addition, a meta-analysis of four longitudinal studies by Yang et al. [29] demonstrated a moderate association between CRP and global cognitive decline. Therefore, CRP seems to be associated with greater age-related cognitive decline. ACEs may also lead to heightened levels of systemic inflammation and may therefore represent a biological pathway linking ACEs to poor adult health outcomes. For instance, a meta-analysis of 25 studies showed that ACE exposure was associated with increased adult inflammatory markers, including CRP [30]. In addition, a recent longitudinal study of older adults found that those reporting three or more ACEs had higher CRP levels over four years [31]. Given that CRP is a biomarker of systemic inflammation that is associated with ACEs, and that systemic inflammation has been identified as a potential risk factor for cognitive decline, CRP may be another potential mediator in the ACEs-to-cognitive decline pathway.

A risk factor is a mediator when it explains the distal impact that another risk factor has on the outcome via its indirect effect [32]. While prior research has linked ACEs to accelerated cognitive decline, the potential roles of depression and inflammation in the ACEs-to-cognitive decline pathway has not yet, to our knowledge, been explored using long-term longitudinal data. Using ELSA data, this study aimed to examine depressive symptoms and inflammation as potential mediators of the ACEs-cognitive decline association. Among this cohort of older adults, we expected that higher cumulative ACEs exposure, retrospectively assessed, would be associated with elevated levels of depressive symptoms and inflammation. Depressive symptoms and inflammation were also hypothesised to represent independent indirect pathways mediating the association between ACEs and cognitive decline.

## Methods

### Study population and design

ELSA is an ongoing prospective cohort study of the health and well-being of older adults. The first wave began in 2002 with a representative sample of 11,391 community-dwelling older adults in England. The core ELSA sample consisted of adults aged 50 + . Younger partners of core members were also eligible to participate. Individuals not living in private households, such as long-term care residents, and those who were unable to consent or respond to the survey questions, for example due to intellectual disability or English language fluency difficulty, were excluded from ELSA.

Data collected included sociodemographic information, depressive symptoms, measures of cognitive function, and blood samples. Follow-up interviews occur every two years and nurse visits, to assess physical functioning and biomarkers, occur every four years. Further information on ELSA sampling and data collection procedures is available elsewhere [33]. The National Health Service Research Ethics Committee approved all waves of ELSA and participants provided informed consent at each wave.

**Fig. 1:**
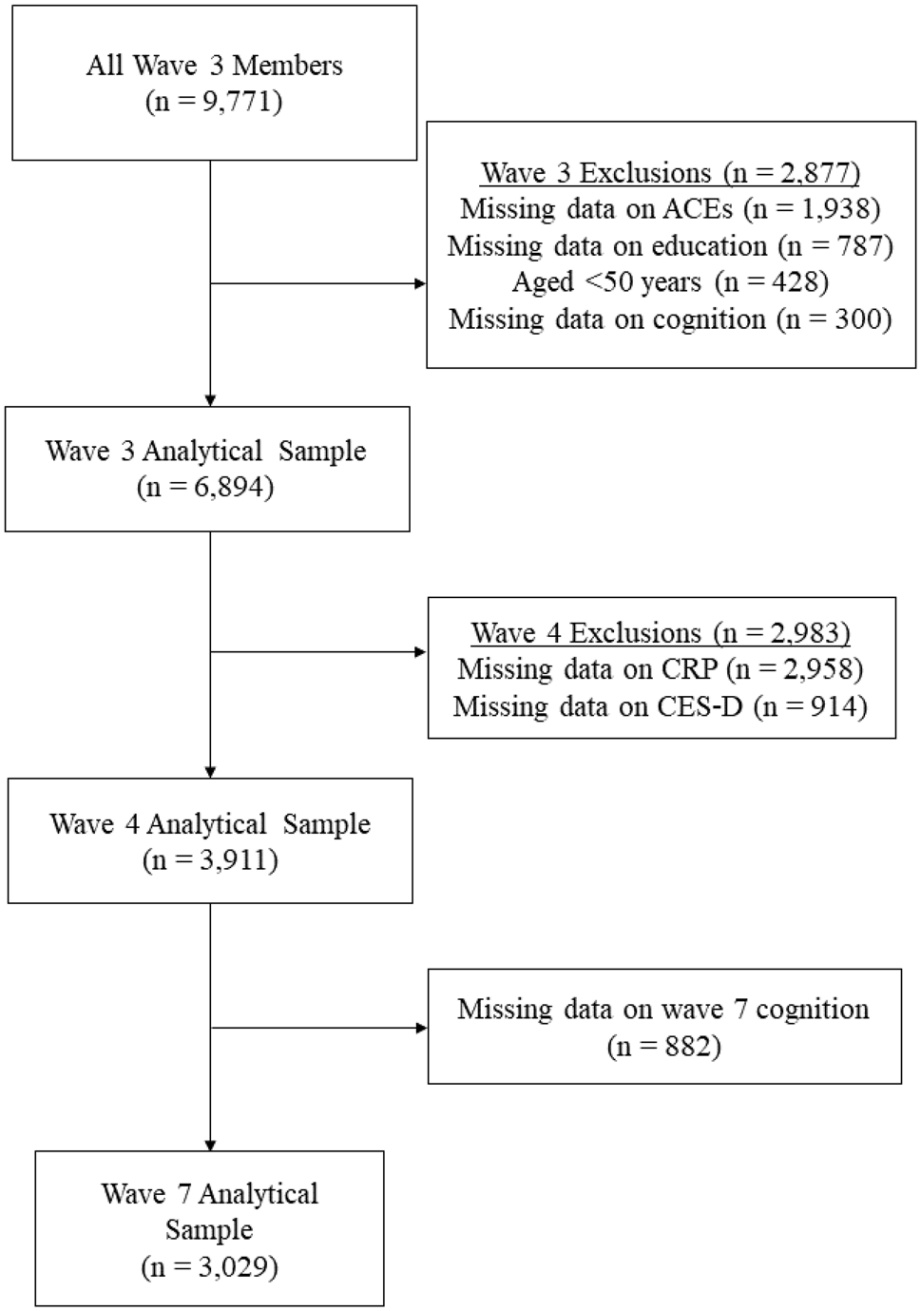
Flowchart illustrating sample selection

Wave 3 of ELSA served as the baseline for the present study because ACEs information was first available at this wave. The current study included participants aged 50 + at baseline with complete ACEs, cognitive function, and socio-demographic information at wave 3 (2006–2007), CRP and depressive symptoms at wave 4 (2008–2009), and cognitive function at wave 7 (2014–2015). Of the 9771 wave 3 participants, 6742 were excluded due to incomplete data or being aged < 50 at baseline. This resulted in an analytical sample of 3029 participants. Figure 1 depicts the sample selection procedure.

### Measures

#### Cognitive function

Each ELSA wave included verbal memory tests, assessing both immediate and delayed recall. Participants were presented with a list of ten common words sourced from the Health and Retirement Study recall tests [34], which were dictated using a voice recording. The number of correct words recalled immediately after hearing the complete list represented immediate memory performance. Following a five-minute delay during which they completed other cognitive tasks, participants were again asked to recall as many of these words as possible, representing their delayed memory performance. Both scores ranged from 0 to 10, with higher scores reflecting better performance. Episodic memory tests, particularly involving delayed recall, have been shown to be accurate and reliable predictors of cognitive decline [35].

Semantic fluency was measured at each wave using an animal naming task. Participants were instructed to name as many animals as possible in one minute. Scores for this task represented a continuous measure of the number of animals mentioned. This ranged from 0 to 56 at wave 3, with higher scores indicating greater performance. This naming task is widely used as a measure of executive function [36].

Temporal orientation was assessed by asking participants to state the current day, date of the month, month, and year. Each accurate answer earned one point. Total scores ranged from 0 to 4, with higher scores indicating better performance. Temporal orientation is routinely included in assessments of global cognitive functioning [37] and involves both semantic abilities and episodic memory [38].

Wave 7 cognitive function was chosen as the main outcome for this study, as this allowed for sufficient time (6–8 years) to investigate associations between baseline measures and cognitive function after the baseline wave, while minimising the effect of attrition on the sample size. Each participant’s general cognitive functioning (g-factor) [39] was calculated from the four cognitive assessments using principal component analysis (PCA). Wave 3 and 7 g-factor scores were saved using a regression approach. These scores were standardised to a mean of zero and standard deviation of 1 with higher scores representing better cognitive function. The g-factor at wave 7 ranged between − 4.22 and 3.36. This approach has previously been used to examine cognitive function assessed by several tasks, in ELSA [40–42] and in other cohort studies [43–45].

#### ACEs

ACEs were reported during the Life History Interview at wave 3 (2006–2007). Participants shared details on their life experiences before age 16. Reported ACEs pertaining to household dysfunction were having lived in a children’s home or with a foster family, having parents who were separated or divorced, having been separated from their mother for six months or longer, having a parent or parents who frequently argued or fought, and having a parent or parents with mental health or substance abuse issues. ACEs pertaining to childhood maltreatment were having been physically abused by a parent and sexual assault, including rape and harassment. A cumulative score (0–8) was calculated representing the total number of ACEs reported by each participant.

#### Depressive symptoms

The 8-item Center for Epidemiologic Studies Depression Scale (CES-D) [46] was used to assess depressive symptoms in all waves. This CES-D Short-Form is widely used and shows good reliability and validity among community-dwelling older adults [47]. Participants responded “yes” (score of 1) or “no” (score of zero) to eight questions on their experience with depressive symptoms over the past week, with six items on depressed affect and two on lack of positive affect. Total scores can range from 0 to 8, with higher scores indicating greater levels of depressive symptoms. The internal consistency of the CES-D scale at wave 4 was good (Cronbach’s alpha = 0.80).

#### C-reactive protein (CRP)

High sensitivity CRP level was assessed using wave 4 (2008–2009) nurse-drawn blood samples. Participants on anticoagulant medication and those with clotting or bleeding disorders were excluded from providing blood samples. Each participant’s blood CRP concentration was modelled as a continuous variable in milligrams per litre (mg/L), with higher values suggesting increased inflammation. CRP values above 3 mg/L are indicative of systemic inflammation [48]. Log-transformation was performed to normalise the highly skewed CRP data before analysis. This method has been employed in previous studies using CRP data in ELSA [49–51].

#### Covariates

Potential confounders of the association between ACEs and cognitive function were adjusted for in all mediation models, including age, sex, education, and wave 3 cognitive g-factor score. ELSA researchers collapsed the age of participants over 90 to protect anonymity. To account for this, age was categorised into 5-year bands, beginning with ‘50–54’ and ending with ‘85 + .’ Sex was reported as male or female and was modelled as a binary variable. Wave 3 participants self-reported their highest educational qualification, ranging from no school qualification to postgraduate degrees. Educational level was modelled as a dichotomous variable in line with previous work [52]: 0–11 years of schooling (up to O-levels) was classified as ‘low’ and 12 + years of schooling (A-levels and above) was classified as ‘high.’ Participants’ cognitive g-factor at wave 3 was calculated using the PCA approached described above. This ranged from − 4.33 to 3.03, with higher scores indicating greater baseline cognition.

In a second set of analyses, lifestyle behaviours were included as additional covariates. Whether wave 3 participants reported currently smoking cigarettes determined their smoking status: ‘current smoker’ or ‘never/former smoker.’ Participants’ reported volume of beer, wine, and spirits consumed in the past week were converted to total units/week [53]. Responses to a questionnaire on physical activity frequency and intensity, adapted from the Whitehall II study [54], were used to categorise physical activity levels as ‘sedentary,’ ‘low,’ ‘moderate,’ or ‘high.’ Nurses measured participants' weight and height at wave 4 to calculate body mass index (BMI).

### Statistical analysis

Analyses were conducted using IBM SPSS Statistics version 26 and the PROCESS macro version 3.5 [55]. Descriptive statistics, including means, standard deviations, and frequencies, were used to assess differences in sociodemographic characteristics, ACEs, and depressive symptoms at baseline (wave 3) between participants included in the analytical sample and those excluded. Statistically significant group differences in categorical variables were assessed using chi-square tests, while continuous variables were evaluated using independent sample *t*-tests. Preliminary associations between the study variables were examined using bivariate Pearson correlations. A hierarchical multiple regression model adjusted for age, sex, and education was performed to investigate the association between ACEs and wave 7 cognitive function.

Statistical mediation was examined by estimating the direct, indirect, and total effects of ACEs on wave 7 cognitive function, and the specific indirect effects of ACEs on wave 7 cognitive function through each mediator (depressive symptoms and CRP), using ordinary least squares regression models with the SPSS PROCESS macro [56]. Direct effects represent the direct impact of the predictor on the outcome variable independent of any mediator; indirect effects are the indirect impact of the predictor on the outcome variable via the mediating variables, and the total effect comprises both direct and indirect effects [57]. Mediation models controlled for potential sociodemographic confounders in a first set of analyses, and additionally controlled for lifestyle behaviours in a second set of analyses. A bootstrapping procedure was used to derive confidence intervals for the indirect effects in each model. Bootstrapping is a nonparametric computational method that involves resampling the complete dataset with replacement data from the original sample to estimate the sampling distribution of the indirect effect [58]. Statistical significance of the indirect effects was determined using 10,000 bootstrap samples to generate a 95% bootstrap confidence interval for all mediation models. A bootstrapped confidence interval that does not overlap with zero indicates statistical significance [56].

Three mediation models were conducted. First, a single mediator model was conducted to test the indirect effect of ACEs on cognition through depressive symptoms. A second single mediator model was conducted to estimate the indirect effect of CRP in the association between ACEs and cognitive function. A final parallel dual-process mediation model was conducted to estimate the indirect effects of ACEs on cognitive function via depressive symptoms and CRP. In the first set of analyses, these models were adjusted for age, sex, education, and baseline cognitive function. The second set of analyses involved further adjusting for smoking, alcohol consumption, physical activity, and BMI, resulting in a reduced sample of 1915.

Sensitivity analyses were conducted to ensure the robustness of the findings. Specifically, sensitivity analyses for the three mediation models, adjusted for sociodemographic factors and baseline cognitive function were performed, using wave 9 (2018–2019) cognitive function as the outcome. This provided four additional years of follow-up from baseline, for a total of 12 years of follow-up on cognition. The wave 9 g-factor was calculated using the same PCA method as for waves 3 and 7 and ranged from − 4.5 to 2.6.

## Results

### Sample characteristics

Baseline characteristics for the included sample compared to participants excluded from analysis are presented in Table 1. Excluded participants were more likely to be older, less educated, and unmarried at baseline, to be of non-white ethnicities, to report more depressive symptoms, and to have lower baseline cognitive function (all *p* < 0.001). These differences persisted when comparing only those with missing ACEs data to those with complete ACEs data. Additionally, only 38.28% of those with missing ACEs data completed wave 7 cognitive tasks.

**Table 1:** Baseline characteristics of the current sample and ELSA wave 3 participants excluded from analysis

	Analytical sample (*n* = 3029)	Wave 3 participants excluded from analysis (*n* = 6742)	*P* value^a^
Age, %			< .001
< 50	0	7.2	
50–54	16.3	16.8	
55–59	25.9	15.1	
60–64	19.3	13.4	
65–69	15.5	11.1	
70–74	12.1	11.6	
75–79	6.9	10.5	
80–84	3.1	7.9	
85 +	0.9	7.2	
Sex, %			0.02
Female	54.8	57.4	
Male	45.2	42.6	
Ethnicity, %			< .001
White	98.1	96.3	
Non-white	1.9	3.7	
Marital status, %			< .001
Married/civil partnership	70.9	65.1	
Unmarried	29.1	34.9	
Educational level, %			< .001
Low	49.3	59.7	
High	50.7	40.3	
ACEs summary score, mean (s.d.)	0.5 (0.9)	0.5 (0.9)	0.3
Baseline cognitive function, mean (s.d.)	0.27 (0.8)	− 0.13 (1.0)	< .001
CES-D total score, mean (s.d.)	1.1 (1.7)	1.56 (2.0)	< .001

*ACEs* adverse childhood experiences; *CES-D* Center for Epidemiologic Studies—Depression scale

^a^Chi-square tests for independence and independent sample *t*-tests were performed to determine whether any significant differences in baseline characteristics existed between the groups

### Bivariate correlations

The associations between ACEs, baseline cognitive function, CRP, depressive symptoms, and cognitive outcomes were explored using Pearson correlations and are presented in Table 2. A weak, positive correlation was observed between the following variables: (1) ACEs and CRP; (2) ACEs and depressive symptoms; and (3) CRP and depressive symptoms. Additionally, a strong, positive correlation was observed between cognitive function at wave 3 and wave 7. There was also a weak, negative correlation found between: (1) CRP and baseline cognitive function; (2) CRP and wave 7 cognitive function; (3) depressive symptoms and baseline cognitive function; and (4) depressive symptoms and wave 7 cognitive function.

**Table 2:** Pearson product-moment correlations between ACEs, CRP, CES-D scores, baseline cognitive function and wave 7 cognitive outcomes

Measure	1	2	3	4	5
1. Total ACEs	–	0.06**	0.11**	0.007	0.023
2. Blood CRP level		–	0.07**	− 0.07**	− 0.07**
3. Total CES-D score			–	− 0.12**	− 0.12**
4. Wave 3 cognitive g-factor				–	0.58**
5. Wave 7 cognitive g-factor					–

*ACEs* adverse childhood experiences, *CRP* C-reactive protein, *CES-D* Center for Epidemiologic Studies—Depression scale

^**^p < 001 (2-tailed)

### Regression and mediation analyses

A hierarchical multiple regression evaluated the association between ACEs and wave 7 g-factor, after controlling for age, sex, and education. Covariates were entered at Step 1, explaining 25.7% of the variance in wave 7 cognition, *F* (3, 3025) = 348.23, *p* < 0.001. After entry of ACEs at Step 2, the overall total variance explained by the model remained unchanged at 25.7%, *F* (4, 3024) = 261.23, *p* < 0.001. ACEs did not make a significant contribution to the variance in wave 7 g-factor, after controlling for age, sex, and education, *R* square change = 0.00, *F* change (1, 3024) = 0.44, *p* = 0.51. ACEs were not found to be a uniquely significant predictor of wave 7 cognitive function (*b* = 0.012, s.e*.* = 0.02, *p* = 0.51).

Unstandardised path coefficients are reported for all mediation analyses. The overall path model adjusted for age, sex, education, and baseline cognitive function testing the indirect effect of ACEs on cognitive function via depressive symptoms was statistically significant. The unstandardised indirect effect of ACEs on cognitive function via the potential mediator depressive symptoms was − 0.007 and was statistically significant (95% CI: − 0.0112, − 0.0040). ACEs were found to be a significant positive predictor of depressive symptoms (*b* = 0.194, s.e*.* = 0.03, *p* < 0.001). Depressive symptoms were, in turn, a significant negative predictor of cognitive function (*b* =  − 0.0370, s.e*.* = 0.007, *p* < 0.001). There was no evidence that ACEs were a significant direct predictor of cognitive function independent of the effect via depressive symptoms (*b* = 0.019, s.e*.* = 0.013, *p* = 0.144), which provided support for the hypothesised mediation model.

Additionally adjusting the model for health behaviour factors yielded similar results. The indirect effect of ACEs on cognitive function via depressive symptoms was weaker ( − 0.0057), but statistically significant (95% CI: − 0.01, − 0.0012). Additionally, a weaker effect size was noted for depressive symptoms as a predictor of cognitive function (*b* =  − 0.03, s.e*.* = 0.012, *p* = 0.012).

The overall path model adjusted for age, sex, education, and baseline cognitive function testing the indirect effect of ACEs on cognitive function via CRP was not statistically significant. The unstandardised indirect effect of ACEs on cognitive function via the potential mediator CRP was  − 0.0005 (95% CI:  − 0.003, 0.001). ACEs were a significant positive predictor of CRP (*b* = 0.0307, s.e*.* = 0.0097, *p* = 0.0015); however, CRP was not found to be a significant predictor of cognitive function (*b* =  − 0.015, s.e*.* = 0.029, *p* = 0.6037). ACEs were not found to be a significant direct predictor of cognitive function in this model (*b* = 0.014, s.e*.* = 0.016, *p* = 0.363). Results of the fully adjusted model were similar to the main analysis, with no notable differences observed.

**Fig. 2:**
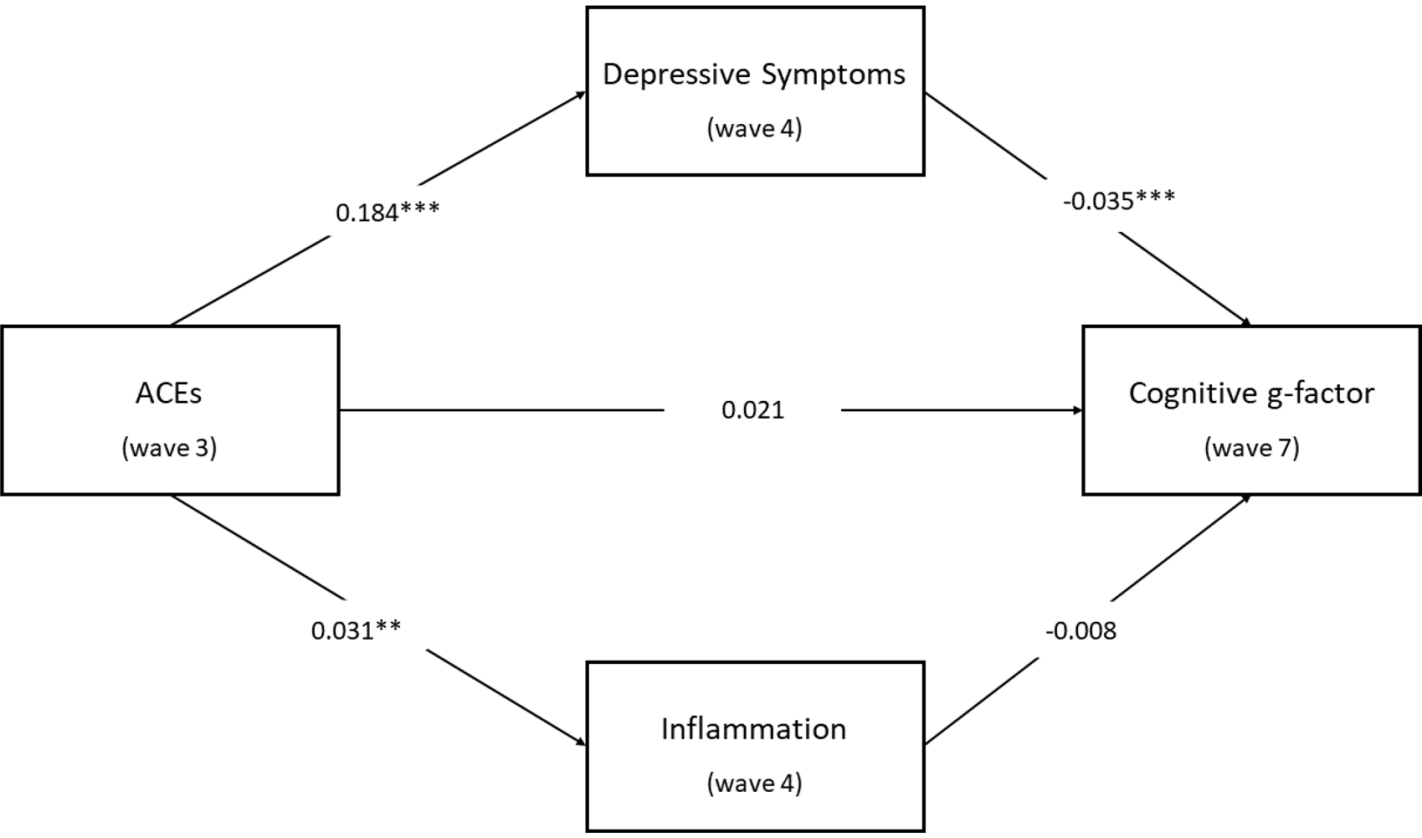
Results of the parallel mediation model testing whether the effect of ACEs on wave 7 cognitive g-factor was mediated by both depressive symptoms and inflammation (CRP). Unstandardised regression coefficients are presented. The indirect effect of ACEs on cognitive function via depressive symptoms ( − 0.006) was statistically significant (95% CI: − 0.01, − 0.003). However, the indirect effect of ACEs on cognitive function via inflammation ( − 0.0002) was not statistically significant (95% CI: − 0.002, 0.0016). The model adjusted for age, sex, educational level, and baseline cognitive function. **p < 0.01; ***p < 0.001

Results demonstrated that the mediation model including both depressive symptoms and CRP as parallel potential mediators, with age, sex, education, and baseline cognitive function as covariates, was statistically significant. Figure 2 illustrates these results. The total indirect effect ( − 0.007) in this model was statistically significant (95% CI: − 0.012, − 0.003). ACEs were a significant positive predictor of depressive symptoms (*b* = 0.184, s.e*.* = 0.034, *p* < 0.001) and remained a significant positive predictor of CRP (*b* = 0.031, s.e*.* = 0.01, *p* = 0.0016). Depressive symptoms were a significant negative predictor of cognitive function (*b* =  − 0.035, s.e*.* = 0.008, *p* < 0.001), whereas CRP was not associated with cognitive function (*b* =  − 0.008, s.e*.* = 0.029, *p* = 0.781). The unstandardised indirect effect of ACEs on cognitive function via the potential mediator depressive symptoms was − 0.006 and was statistically significant (95% CI: − 0.01, − 0.003). The unstandardised indirect effect of ACEs on cognitive function via the potential mediator CRP was − 0.0002 and was not statistically significant (95% CI: − 0.002, 0.0016). ACEs were not shown to be a significant direct predictor of cognitive function (*b* = 0.021, s.e*.* = 0.016, *p* = 0.178), suggesting that the role that ACEs have in cognitive function in this model may be distal via depressive symptoms.

In the fully adjusted model, the total indirect effect ( − 0.0046) became non-significant (95% CI: − 0.0011, 0.0007). Depressive symptoms had a reduced effect size as a predictor of cognitive decline compared to the main analysis (*b* = 0.029, s.e*.* = 0.012, *p* = 0.0129).

Results of the sensitivity analyses using the wave 9 cognitive g-factor as the outcome, with age, sex, education, and baseline cognitive function as covariates, were consistent with the results presented in the original models. Table 3 (see supplemental file) details the results of the dual-path model sensitivity analysis alongside the original parallel mediation model results.

## Discussion

The present study investigated depressive symptoms and CRP as potential mediators of the association between ACEs and later cognitive function among older adults living in England. Retrospectively reported ACEs were found to be significantly associated with depressive symptoms in later life, which is consistent with findings from previous studies demonstrating a graded exposure–response relationship between ACEs and adult depressive symptoms [11, 19, 59, 60] including during later life [21–23]. The hypothesis that depressive symptoms might be an indirect mechanism linking ACEs to cognitive decline was supported. ACEs at wave 3 significantly predicted increased depressive symptoms at wave 4, which in turn had a significant negative effect on cognitive function at wave 7. These results support findings by Davis et al. [24] demonstrating that depressive symptoms mediated the association between child maltreatment and adult cognition. Adjusting for health behaviours and BMI reduced the strength and significance of depressive symptoms as a predictor of cognitive decline, suggesting that these may represent additional risk factors for cognitive decline, in line with the report by Livingston and colleagues [61]. Notably, the association between ACEs and later cognitive function was not statistically significant in this sample, which was inconsistent with previous findings. ACE exposure may not have been a significant direct predictor of cognitive decline in this study due to additional unexamined confounders which future studies may help to elucidate.

ACEs were found to be positively associated with CRP. This finding is consistent with prior research linking ACE exposure to elevated adult CRP [30, 31]. Higher CRP levels were shown to be correlated with lower baseline and wave 7 cognition, which supports prior research demonstrating an inverse association between CRP and cognitive performance in older adults [27–28]. However, this study found no evidence to support the hypothesis that ACEs are linked to an increased risk of cognitive decline via elevated CRP. Although previous findings have supported CRP as a risk factor for cognitive decline [62–63], CRP has shown only a marginal association with global cognitive decline in prior longitudinal research [29]. Davis et al. [24] found that inflammation mediated the link between child maltreatment and poor cognitive function. The use of interleukin-6 (IL-6) as an inflammatory biomarker rather than CRP may account for these disparate findings, though further investigation is needed.

Findings from this study are partially in keeping with the biological embedding of childhood adversity model which posits that ACE exposure may cause biological alterations that have long-term physical and mental health consequences [64]. Children who are exposed to adversity without a supportive adult caregiver may develop "toxic stress," which impairs the immunological and neuroendocrine systems [65]. Raymond et al. [66] note that ACE exposure implicates hypothalamic–pituitary–adrenal (HPA) axis dysregulation, impacting the development of the hippocampus, prefrontal cortex, and amygdala and likely altering underlying cognitive processes, which in turn increases vulnerability to certain adult mental illnesses. This model is supported by research demonstrating that toxic stress increases the risk of permanent changes in brain architecture and gene regulation, which are linked to adult-onset conditions including depression [67]. Emotion regulation deficits, rumination, threat reactivity, and impulsive behaviours have been linked to ACE exposure and are also associated with an increased risk of depression [68]. Depression may in turn contribute to cognitive decline, possibly by disrupting the HPA axis or through interaction with genetic predispositions [69]. The potential mediating role of CRP in the ACEs-cognition decline association should be examined in further research.

Strengths of this study include the use of longitudinal data from a large, nationally representative sample of older adults in England. The older age of this sample corresponds to the life stage during which cognitive decline is most likely to emerge. Another strength of this study is that the mediation models were adjusted for several potential confounders and sensitivity analyses were performed.

Certain limitations must also be considered when interpreting the results of this study. ELSA data includes only traditional ACEs, as researched by Felitti et al. [11]. Recent research has examined a broader spectrum of ACEs, which may more accurately represent childhood adversity [70], including community violence, economic hardship, and bullying [71, 72]. ACEs were reported retrospectively, which may limit their validity due to recall or response biases [73, 74]. Adult recollections of childhood stressors may be inaccurate due to memory difficulties, current depressed mood [75, 76], or agreeableness [77]. A more thorough battery of standardised tests, encompassing more cognitive domains [78], would additionally have allowed for a more reliable assessment of cognitive function in this study.

A considerable number of ELSA participants were excluded from analysis due to missing data. The Life History Interview, which gathered information on ACEs in ELSA, was optional, which may have resulted in selection bias. Comparatively, the analytical sample had lower levels of depressive symptoms and generally high cognitive function at baseline. This may have resulted in conservative estimates of the effects of these variables on cognitive outcomes. The final sample was disproportionately white and highly educated which may limit the representativeness of the sample and generalisability of the findings.

More longitudinal research with diverse samples is required to further investigate the biological and psychological pathways linking ACEs to cognitive decline. Future research may also benefit from examining a broader range of ACEs and determining whether certain subtypes of ACEs affect health outcomes differently. Different inflammatory biomarkers including IL-6 should be further studied, as prior research has found different inflammatory biomarkers to be associated with cognitive function [24, 79, 80]. Given that social engagement has been shown to reduce the risk of cognitive decline [81], future studies using ELSA data might examine the potential role of this variable in the ACEs-cognitive decline association. Datasets containing detailed data on nutrition could also be employed to examine the potential role of eating behaviour in the association between ACEs and cognitive function. The current study investigated only the stationary effects of depressive symptoms and inflammation on cognitive decline. However, more dynamic models accounting for a potential temporal association between depressive symptoms and inflammation could be examined [23]. This method may provide additional insight into how these two potential mediators interact over time to affect cognitive outcomes.

Using longitudinal data and mediation analysis, this study adds to the growing body of evidence linking ACEs with poor physical, cognitive, and mental health in adulthood. Specifically, findings indicate that depressive symptoms may represent a pathway linking ACEs with cognitive decline in later life. ACEs were also shown to predict adult systemic inflammation, though this did not mediate the association with cognitive function. Findings suggest that public health screening, prevention, and intervention methods used to mitigate the effects of ACEs during childhood may be a key strategy towards improving health throughout the life course. The potential worldwide increase in ACEs including abuse and neglect during the COVID-19 pandemic [82] may heighten the pertinence of this consideration. Early intervention programmes targeting known mediators of the association between ACEs and depression (e.g. negative cognitive style, lack of social support, and emotional dysregulation [83]), as well as those promoting the protective factor of resilience [84], may represent promising avenues for reducing the risk of ACE-related negative health outcomes.

## Supplementary Information

Below is the link to the electronic supplementary material.

Supplementary file1 (DOCX 22 KB)
