# Supplementary material for: Adverse childhood experiences and cognitive function in adulthood: examining the roles of depressive symptoms and inflammation in a prospective cohort study
Source: Soc Psychiatry Psychiatr Epidemiol. 2022 Jun 26;57(12):2367–77. doi: 10.1007/s00127-022-02315-w (PMC9244111; doi:10.1007/s00127-022-02315-w)
Supplement: Supplementary file 1 — Supplementary file1 (DOCX 22 KB) [file 127_2022_2315_MOESM1_ESM.docx]

**Supplementary Information**

Table 3 Results of the sensitivity analysis for the dual mediation model

|  | **Wave 7 Dual Mediation Model adjusted for sociodemographic factors and baseline cognitive function**  ***(n = 3,029)*** | **Wave 7 Dual Mediation Model adjusted for additional health factors**  ***(n = 1,915)*** | **Wave 9 Sensitivity Analysis adjusted for sociodemographic factors and baseline cognitive function**  ***(n = 2,343)*** |
| --- | --- | --- | --- |
| Total indirect effect of ACEs on cognitive function via depressive symptoms and CRP | b = -0.007, s.e. = 0.002 (95% CI: -0.012, -0.003) | b = -0.0046, s.e. = 0.003 (95% CI: -0.011, 0.001) | b = -0.005 , s.e. = 0.003 (95% CI: -0.011,  -0.0003) |
| Direct effect of ACEs on depressive symptoms | b = 0.184, s.e. = 0.034, p < .001 (95% CI: 0.118, 0.251) | b = 0.191, s.e. = 0.038, p < .001 (95% CI: 0.115, 0.266) | b = 0.146, s.e. = 0.037, p < .001 (95% CI: 0.073, 0.218) |
| Direct effect of ACEs on CRP | b = 0.031, s.e. = 0.01,  p = 0.0016 (95% CI: 0.012, 0.05) | b = 0.027, s.e. = 0.012,  p = 0.022 (95% CI: 0.004, 0.05) | b = 0.041, s.e. = 0.011, p < .001 (95% CI: 0.019, 0.062) |
| Direct effect of depressive symptoms on cognitive function | b = -0.035, s.e. = 0.008, p < .001 (95% CI: -0.051, -0.019) | b = -0.029, s.e. = 0.012, p = 0.013 (95% CI: -0.052, -0.006) | b = -0.031, s.e. = 0.011, p = 0.004 (95% CI: -0.051, -0.01) |
| Direct effect of CRP on cognitive function | b = -0.008, s.e. = 0.029, p = 0.781 (95% CI: -0.065, 0.049) | b = 0.038, s.e. = 0.038, p = 0.323 (95% CI: -0.037, 0.112) | b = -0.011, s.e. = 0.036, p = 0.752 (95% CI: -0.081, 0.059) |
| Indirect effect of ACEs on cognitive function via depressive symptoms | b = -0.006, s.e. = 0.002 (95% CI: -0.01,  -0.003) | b = -0.006, s.e. = 0.003 (95% CI: -0.011,  -0.001) | b = -0.005 , s.e. = 0.002 (95% CI: -0.01, -0.001) |
| Indirect effect of ACEs on cognitive function via CRP | b = -0.0002 , s.e. = 0.001 (95% CI:  -0.002, 0.0016) | b = 0.001, s.e. = 0.001 (95% CI:  -0.001, 0.004) | b = -0.0005 , s.e. = 0.002 (95% CI:  -0.004, 0.002) |
| Direct effect of ACEs on cognitive function | b = 0.021, s.e. = 0.016, p = 0.178 (95% CI: -0.01, 0.051) | b = 0.022, s.e. = 0.02, p = 0.278 (95% CI: -0.017, 0.06) | b = -0.0026,  s.e. = 0.019, p = 0.892 (95% CI: -0.04, 0.035) |
